# Interaction between Corneal and Internal Ocular Aberrations Induced by Orthokeratology and Its Influential Factors

**DOI:** 10.1155/2017/3703854

**Published:** 2017-08-06

**Authors:** Qingzhong Chen, Min Li, Ying Yuan, Rao Me, Yunjie Yu, Guangsen Shi, Bilian Ke

**Affiliations:** Department of Ophthalmology, Shanghai General Hospital, Shanghai Jiao Tong University School of Medicine, Shanghai, China

## Abstract

**Purpose:**

To investigate the interaction between corneal, internal, and total wavefront aberrations (WAs) and their influential factors during orthokeratology (OK) treatment in Chinese adolescents.

**Methods:**

Thirty teenagers (*n* = 30 eyes) were enrolled in the study; spherical equivalent refraction (SE), corneal curvature radius (CCR), central corneal thickness (CCT), WAs, and the difference in limbal transverse diameter and OK lens diameter (ΔLLD) were detected before and after one-month OK treatment. Every component of WAs was measured simultaneously by iTrace aberrometer. The influential factors of OK-induced WAs were analyzed.

**Results:**

SE and CCT decreased while CCR increased significantly (*P* < 0.01). Higher-order aberrations (HOAs), Spherical aberrations (SAs), and coma increased significantly (*P* < 0.01). Corneal horizontal coma (Z3^1^-C) and corneal spherical aberrations (Z4^0^-C) increased (*P* < 0.01). The HOAs, coma, SAs, Z3^1^-C, Z3^1^-T, Z4^0^-C, and Z4^0^-T were positively correlated with SE and CCR (*P* < 0.01). Z3^−1^-C showed negative correlations with (ΔLLD) and positive correlations with SE (*P* < 0.05).

**Conclusions:**

The increase in OK-induced HOAs is mainly attributed to Z3^1^ and Z4^0^ of cornea. Z3^−1^ in the internal component showed a compensative effect on the corneal vertical coma. The degree of myopic correction and increase in CCR may be the essential influential factors of the increase in Z3^1^ and Z4^0^. The appropriate size of the OK lens may be helpful to decrease OK-induced vertical coma.

## 1. Introduction

Myopia has become a significant public health problem worldwide, especially in some Asian countries, where it presents a massive socioeconomic burden on society [1]. The outcome may be attributed to the unprecedented increase in the prevalence and the earlier age of onset of myopia [2–4]. Orthokeratology (OK) is a reversible procedure for temporally correcting low to moderate degrees of myopia [4, 5]; numerous previous studies have confirmed the potential effect of overnight OK for retarding the progression of myopia [6, 7]. However, OK causes an increase in higher-order aberrations (HOAs), which were first reported by Joslin et al. [8] and are well demonstrated by other researchers [9–13].

HOAs and myopia have been studied for a long time. Zhang et al. [14] and Hiraoka et al. [15] suggested that the increase in HOAs might be a risk factor for myopia progression. In addition, it was reported that HOAs was correlative with age [16], cause in young eyes, corneal aberrations could be compensated by internal aberrations. Such a compensatory effect could also be found in patients after corneal laser refractive surgery [17]. The OK-induced HOAs usually present an increase in coma-like aberrations and a positive shift in spherical aberrations (SAs) [10, 12], but the interaction between corneal and internal aberrations in children and adolescents who are wearing OK lenses is still unknown.

The profile of OK-induced changes relative to total ocular aberrations is similar to changes in corneal aberrations [9, 11], which indicates that the increase in ocular aberrations by OK may be predominantly attributable to the reshaping of the corneal surface. The internal aberrations are determined by subtracting corneal aberrations from total aberrations; therefore, accurate measurement of corneal and total aberrations is essential. We used the iTrace aberrometer (Tracey Technologies, Houston, TX, USA) to measure specific changes in every components (corneal, internal, and total) of wavefront aberrations (WAs) in the study, cause compared to other aberrometers, iTrace aberrometers showed the best reproducibility for corneal aberrations [18].

Several influential factors during OK treatment may contribute to the change in OK-induced HOAs, such as myopic spherical equivalent (SE), corneal curvature radius (CCR), central corneal thickness (CCT), limbal transverse diameter (Limbal-D), and lens diameter. These influential factors of the OK-induced WAs were also discussed in the present study.

## 2. Materials and Methods

### 2.1. Tests Performed

Thirty patients (*n* = 30 eyes) were enrolled in the study; the inclusion criteria for this study were as follows: age range from eight to eighteen years, myopic spherical equivalent less than −6.00 D, regular forms of astigmatism less than −1.00 D, best spectacle-corrected visual acuity of 20/20 or better, mean keratometry reading between 40.00 and 46.00 D, absence of any ocular and systemic diseases, and no previous treatment with overnight OK. The study adhered to the tenets of the Declaration of Helsinki and was approved by the Ethics Committee of Shanghai Jiaotong University. Informed consent was obtained from all the patients and from at least one of their parents with full understanding of the purpose, protocol, and potential risk of the study.

Each patient underwent a comprehensive baseline examination following the sequence: manifest refraction, best-corrected visual acuity (BCVA), corneal topography, aberrometry, and CCT measurement. The corneal topography, Limbal-D, and aberrometry were accessed using the iTrace system (Trace Technologies, Houston, USA) that is uniquely designed to combine Placido corneal topography with a ray-tracing aberrometer to measure quality of vision in a patient; this enables to analyze the entire eye by each component. The corneal aberrations were obtained with the Vista attachment of the wavefront aberrometer, and aberrations in the internal optics were calculated by subtracting the aberrations of the cornea from those of the entire eye measured by the ray-tracing aberrometer using a built-in program [19]. The Zernike coefficient of vertical coma (Z3^−1^), horizontal coma (Z3^1^) and spherical aberrations (Z4^0^ and Z6^0^) of the cornea, internal optics, and the ocular aberrations of the entire eye were evaluated. The root mean square (RMS) of total aberrations (Total), lower-order aberrations (LOAs), HOAs, coma, and SAs were obtained for a 4 mm pupil diameter. Each measurement was repeated at least five times and the three best-focused results were selected and averaged. To minimize the influence of diurnal variation, all measurements were conducted between 9 and 12 AM, and patients were requested to attend the examinations from 2 to 4 hours after lens removal. Additionally, to minimize systematic bias, only the right eye of each patient was included in the study.

### 2.2. Contact Lens Used

After the baseline examination, the OK lens (E&E Optics Ltd, Hong Kong, China) was selected to be fitted for the enrolled patients on the basis of the instructions recommended by the manufacturer. The OK lenses used in this study were of a 4-zone reverse geometry design, with the Boston XO material having the gas permeability of 100 × 10^−11^ (cm^2^  ×  mLO_2_)/(s × mL × mmHg). The back optical zone radius (BOZR) and the alignment curve (AC) radius were determined by the cycloplegic manifest refraction, corneal flat-K, and corneal eccentricity. The overall diameter of the lens was 10.3 to 10.9 mm and the optic zone diameter was 6.0 mm. The central thickness was 0.22 mm. Lens fitting was evaluated by slit-lamp biomicroscopy with corneal fluorescein staining tests 1 and 2 hours after delivery of the lens. The lens should be well centered on the cornea and move approximately 1 mm on a blink to meet a fit to be acceptable. Each patient was instructed to wear the selected OK lens at least 8 hours during the night and to remove it directly after eye opening. The follow-up was performed one week after the first visit and one month after OK wear. The patients were required to come back for all the above-mentioned examinations, among them, the ray-tracing aberrometry. The value of Limbal-D minus lens diameter was also calculated and defined as ΔLLD. By topography, all patients showed typical Bulls eye pattern on axial corneal maps and an ideal centration of the treatment zone on tangential subtractive maps. All the exams were performed by the same technician who was experienced in conducting each measurement of the study.

### 2.3. Statistical Analysis

The measured data were presented as mean ± standard deviation (SD). Kolmogorov-Smirnov test was used for Normality Test. The baseline data and one-month posttreatment data were compared using the paired *t*-test (normally distributed) and the Wilcoxon signed-rank test (not normally distributed) for the eye parameters and the aberrations. Pearson coefficients and Spearman coefficients were performed to identify the influential factors with a statistically significant contribution to OK-induced WAs. All statistics were performed using SPSS 23 (IBM Corporation, USA). *P* < 0.05 was regarded as statistically significant.

## 3. Results

### 3.1. The Change in Ocular Measurement Readings after One-Month Overnight OK

All of the thirty patients successfully completed the one-month follow-up examinations. There were eighteen females and twelve males in the study; their age was between eight and sixteen years (11.73 ± 2.27, mean ± SD) and the mean ocular refraction was −3.44 ± 1.47 D. After overnight OK, all the patients reached an uncorrected visual acuity of 20/20. Likewise, the CCT was decreased from 548.17 ± 35.05 *μ*m to 532.00 ± 35.72 *μ*m (*P* < 0.01, paired *t*-test). In contrast, the CCR was increased noticeably from 7.85 ± 0.43 mm to 8.52 ± 0.48 mm (*P* < 0.01, paired *t*-test) after one month with OK.

### 3.2. RMS Values of Total, LOAs, HOAs, Coma, and SAs for a 4.0 mm Pupil Size at the Baseline and after One-Month Overnight OK (Figure 1())

RMS Total, LOAs and SAs were normally distributed (*P* = 0.20, *P* = 0.20, and *P* = 0.05, resp.), while HOAs and coma were not normally distributed (*P* < 0.01). The mean Total RMS decreased from 2.26 ± 0.91 *μ*m at baseline to 1.23 ± 0.71 *μ*m after one month of OK (*P* < 0.01). There was also statistically significant decrease of LOA RMS from 2.24 ± 0.93 *μ*m at baseline to 1.12 ± 0.72 *μ*m after one month of OK (*P* < 0.01). In contrast, the mean HOA increased significantly from 0.18 ± 0.19 *μ*m to 0.49 ± 0.25 *μ*m (*P* < 0.01). The mean Coma and SA increased remarkably from 0.12 ± 0.19 *μ*m to 0.36 ± 0.23 *μ*m (*P* < 0.01) and 0.05 ± 0.06 *μ*m to 0.19 ± 0.13 *μ*m (*P* < 0.01), respectively.

### 3.3. The Components of Coma and SAs for a 4.0 mm Pupil Size (Figure 2())

Wilcoxon signed-rank test was used because the Zernike coefficient readings were not normally distributed (*P* < 0.05, Kolmogorov-Smirnov test). The Zernike coefficient of corneal vertical coma (Z3^−1^-C) increased from 0.01 ± 0.09 *μ*m at baseline to 0.09 ± 0.24 *μ*m after one-month OK treatment, but the internal vertical coma (Z3^−1^-I) showed a decrease from 0.04 ± 0.22 *μ*m to −0.02 ± 0.12 *μ*m, which led to only a slight increase in the total ocular vertical coma (Z3^−1^-T) from 0.06 ± 0.25 *μ*m to 0.07 ± 0.22 *μ*m; however, these differences were not statistically significant (*P* = 0.20). The corneal horizontal coma (Z3^1^-C) increased dramatically from 0.00 ± 0.10 *μ*m to 0.20 ± 0.33 *μ*m (*P* < 0.01). The change in the total ocular horizontal coma (Z3^1^-T) was similar to Z3^1^-C (from 0.03 ± 0.08 *μ*m to 0.24 ± 0.23 *μ*m; *P* < 0.01). The internal horizontal coma (Z3^1^-I) did not change significantly (*P* = 0.70). The corneal SAs (Z4^0^-C) increased significantly from 0.07 ± 0.04 *μ*m to 0.18 ± 0.10 *μ*m (*P* < 0.01), while the internal SAs (Z4^0^-I) shifted positively from −0.02 ± 0.04 *μ*m to 0.01 ± 0.10 *μ*m even though the change was not statistically significant (*P* = 0.07); the total ocular SAs (Z4^0^-T) increased from 0.05 ± 0.05 *μ*m to 0.19 ± 0.13 *μ*m (*P* < 0.01). The corneal and total ocular higher-order SAs (Z6^0^-C, Z6^0^-T) did not change significantly after one-month follow-up (*P* = 0.67, *P* = 0.05, resp.), but the internal (Z6^0^-I) shifted positive from −0.01 ± 0.03 *μ*m to 0.01 ± 0.03 *μ*m (*P* < 0.01).

### 3.4. The Relationship between the Ocular Parameter Readings (SE, CCR, and ΔLLD) and RMS and Zernike Coefficients Induced after One-Month Overnight OK (Figures 3()–5())

**Figure 3:**
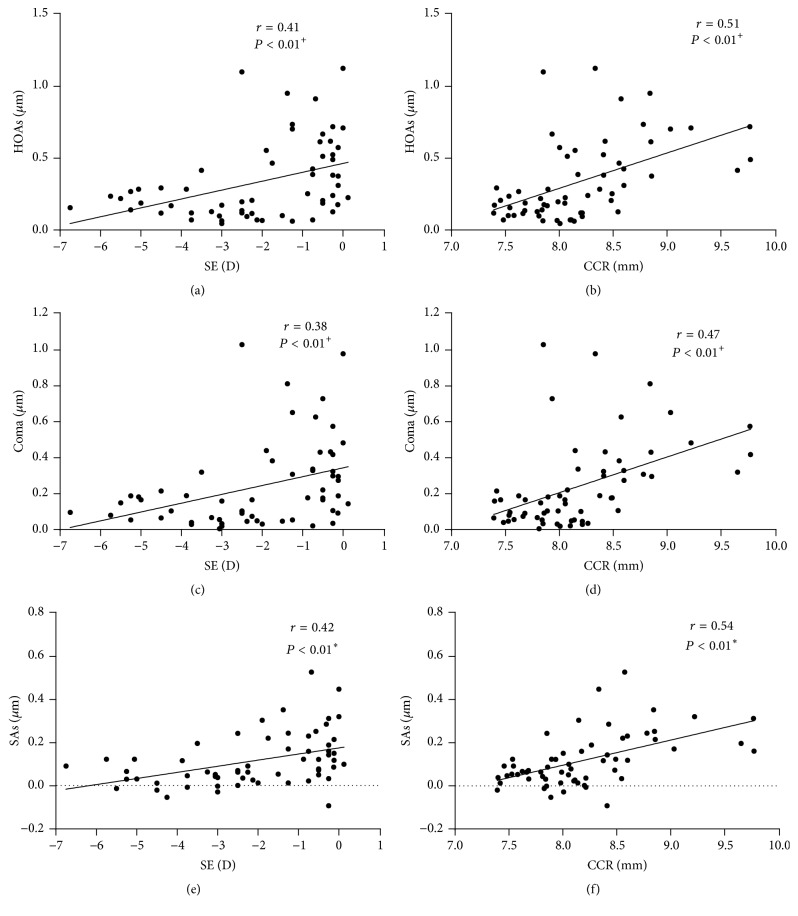
Correlation between HOAs (higher-order aberrations), coma, and SAs (spherical aberrations) with the spherical equivalent (SE) and the CCR (corneal curvature radius) after one-month orthokeratology treatment; ^+^Spearman coefficients; ^*∗*^Pearson coefficients, *n* = 30 eyes.

**Figure 4:**
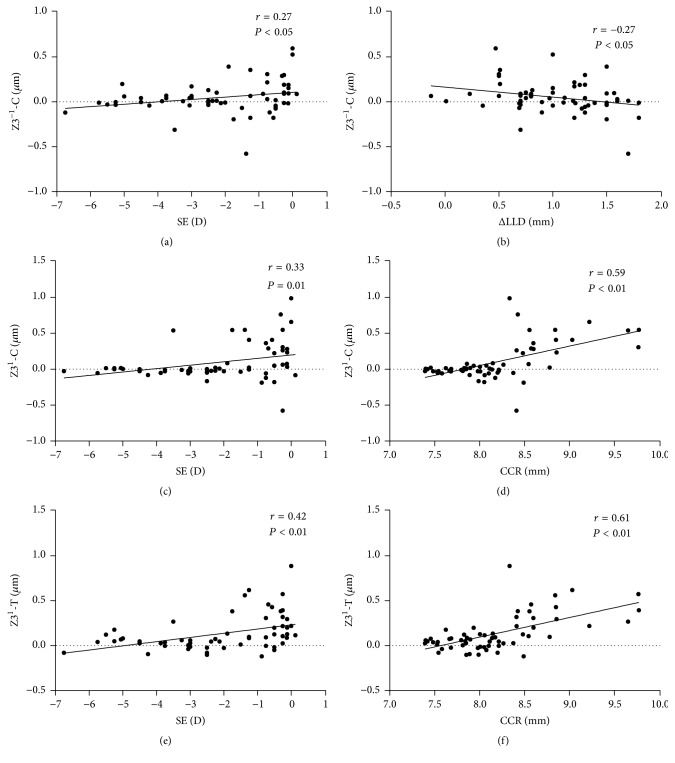
Correlation between Z3^−1^-C, Z3^1^-C, Z3^1^-T, and ΔLLD (difference between limbal transverse diameter and lens diameter), spherical equivalent (SE), and CCR (corneal curvature radius) after one-month orthokeratology treatment; Spearman coefficients; *n* = 30 eyes.

**Figure 5:**
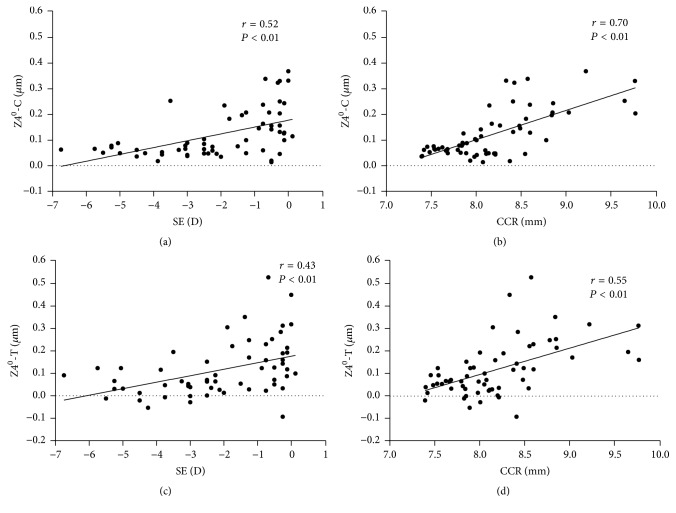
Correlation between Z4^0^-C and Z4^0^-T with the spherical equivalent (SE) and CCR (corneal curvature radius) after one-month orthokeratology treatment. Spearman coefficients; *n* = 30 eyes.

**Figure 6:**
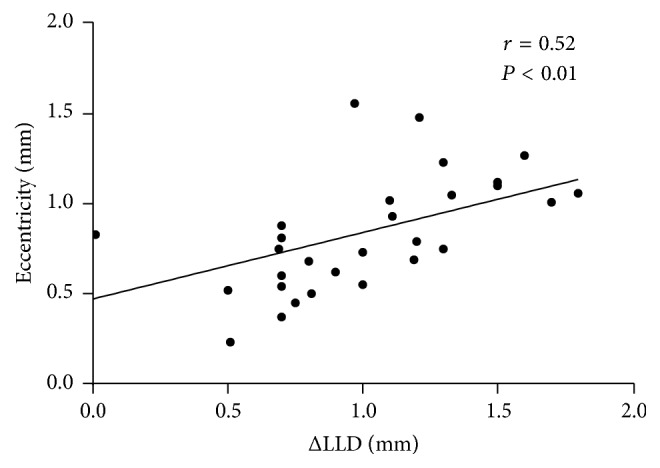
The correlations between eccentricity and ΔLLD (difference between limbal transverse diameter and lens diameter) after one-month orthokeratology treatment. Spearman coefficients.

To assess the influential factors of OK-induced WAs, Pearson coefficients and Spearman coefficients were used for the correlation between the ocular parameter readings and WAs as well as for the correlation between those and the Zernike coefficients. The HOAs were significantly correlated with SE (*r* = 0.41, *P* < 0.01) and CCR (*r* = 0.51, *P* < 0.01). The Coma and SAs showed significant correlations with SE (*r* = 0.38, *r* = 0.42, *P* < 0.01) and CCR (*r* = 0.47, *r* = 0.54, *P* < 0.01, Figure 3()). Z3^−1^-C showed significant correlations with ΔLLD (*r* = −0.27, *P* = 0.03) and SE (*r* = 0.27, *P* = 0.04). Z3^1^-C and Z3^1^-T showed significant correlations with SE (*r* = 0.33, *P* = 0.01, *r* = 0.42, *P* < 0.01) and CCR (*r* = 0.59, *r* = 0.61, *P* < 0.01, Figure 4()). Z4^0^-C and Z4^0^-T also showed significant correlations with SE (*r* = 0.52, *r* = 0.43, *P* < 0.01) and CCR (*r* = 0.70, *r* = 0.55, *P* < 0.01, Figure 5()). We further analyzed the correlation between SE and CCR and found a positive correlation (*r* = 0.542, *P* = 0.001). We also found that, after one month of OK treatment, the average eccentricity of TZ was 0.83 ± 0.32 mm. The eccentricity of TZ was positively correlated with the value of ΔLLD (*r* = 0.524, *P* = 0.003, Figure 6()).

## 4. Discussion

Overnight OK has been used to correct low to moderate myopia for many years; over the last few years, a number of clinical studies have reported that overnight OK increased HOAs [20–24]. The OK-induced WAs usually presented as a positive shift of SAs and an increase in coma aberrations [12, 25]. Even though OK-induced HOAs have been studied for quite some time, the interaction among each component of HOAs after OK remains ambiguous. Gifford et al. [12] investigated the relationship between OK-induced changes to the anterior corneal surface and ocular aberrations, but they did not segregate into constituent Zernike components; moreover, small sample size and short-term follow-up (7 days) also limited the results. Lian et al. [13] have investigated the relationship between changes in corneal reshaping and induced WAs; however, they analyzed only the association between corneal thickness and WAs. It is well known that the ocular WAs are composed of corneal aberrations and internal aberrations. The interaction between corneal and internal aberrations after OK and their contributions to the eventual change in WAs needed to be clarified. In addition, the influential factors of each component of aberrations after OK also needed to be clarified.

It has been confirmed that most optical aberrations stabilize within the first week after beginning OK [12, 26]. On the basis of the previous results, we conducted a 30-day follow-up in the current study to ensure that the WAs status was already stable after OK. Our results showed that the change in Total RMS was extremely similar to the change in LOAs (2.26 *μ*m to 1.23 *μ*m and 2.24 *μ*m to 1.12 *μ*m); further analysis showed that Total RMS decreased by 1.03 *μ*m from the baseline examination to one-month follow-up, while LOAs decreased by 1.12 *μ*m. LOAs presented a greater decrease than Total RMS due to the increase in HOAs. Furthermore, Coma and SA showed approximately 3-fold and 4-fold increase, respectively, after one month of OK wear, which were consistent with the results of Hiraoka et al. [22]. Lian et al. [13] obtained a similar result of coma (2.6-fold) and a greater change in SAs (7.3-fold), which may be because they measured the WAs over 6 mm pupil diameter. After myopia correction, HOAs (coma + SAs) showed a positive correlation with the SE, which coincided with those of previous studies [15, 27]. It is well known that myopic OK applies positive pressure at the center of the cornea and negative pressure in the midperiphery under the steeper secondary “reverse” curve. Consequently, it produces a flattened central treatment zone that corrects the myopic refractive error by reducing corneal power [13]. The corneal reshaping is sure to lead to a significant increase in the CCR. The positive correlation between SE and CCR indicated that the level of achieved myopic correction by OK contributed to the change in aberrations by exaggerating the CCR. Even though we considered Lens-D, Limbal-D, and ΔLLD as the potential influential factors of OK-induced WAs, no significant correlations were found between them.

To clarify the interaction of each component of WAs affected by overnight OK, we analyzed the Zernike coefficient of Z3^−1^, Z3^1^, Z4^0^, and Z6^0^ of the cornea, the internal optics, and the total ocular aberrations. We found that Z3^−1^-C increased significantly from 0.01 *μ*m to 0.09 *μ*m after one month of OK, but Z3^−1^-T barely increased. Because we also found a negative shift of Z3^−1^-I at the one-month examination, which indicated a compensative effect of the ocular, it meant that an ocular adaptation response toward neutralizing induced positive corneal vertical coma by OK existed. We also found a dramatic increase in corneal horizontal coma (Z3^1^-C) at one-month follow-up, but there was no compensation in Z3^1^-I; the increase in Z3^1^-C contributed to the change in Z3^1^-T. It seemed that overnight OK caused a greater effect on horizontal coma, mostly by increasing the horizontal coma of the cornea. Gifford et al. [12] found that there may be an ocular adaptation response in the internal SAs to neutralize the induced positive SAs; however, in the present study, we could not find the compensative effect of internal Z4^0^ and Z6^0^. After one month, Z4^0^-C increased significantly while Z6^0^-C remained almost unchanged. The increase in Z4^0^-C directly led to a growth of Z4^0^-T. We also noticed that both Z4^0^-I and Z6^0^-I showed a positive shift at the one-month examination, which indicated that the internal SAs could not compensate for the change in corneal SAs during OK wear.

Similar to the RMS of WAs, SE and CCR were also correlated with the Zernike coefficients. As the main components of OK-induced HOAs, Z3^1^-C and Z4^0^-C were both positively correlated with SE and CCR. In other words, the amount of myopic correction by OK and the level of corneal flattening were closely related to OK-induced WAs, and they manifested mainly as an increase in horizontal coma and Z4^0^. Even though vertical coma (Z3^−1^) remained almost unchanged due to the compensation of the internal component, Z3^−1^-C showed a remarkable increase after one month of OK. The increase in Z3^−1^-C was positively correlated with SE and surprisingly negatively correlated with ΔLLD. The unexpected negative correlation between Z3^−1^-C and ΔLLD meant the greater the ΔLLD (or the lower the Lens-D) is, the lower the induction in Z3^−1^-C was observed. Although statistically significant, this correlation was not strong (*r* = −0.27). Stillitano and coauthors [10] found different directionality of induced horizontal coma between right and left eyes that was reported as a subclinical temporal decentration. Although at the present study only right eyes were enrolled, the induction of horizontal coma showed remarkable after OK, reaffirming that some decentration mainly to lateral side occurred as well. It is well known that Coma and SAs are prone to be influenced by the eccentricity and tilting of the optic zone or lens [28, 29]. We hypothesized that limbal diameter and lens diameter may be potential influential factors of HOAs generated from OK wear. Seldom studies have noticed the difference between limbal transverse diameter and lens diameter. The Lens-D is determined using the Limbal-D reading of patients and is usually 0.5 mm–1.5 mm less than Limbal-D; there are no precise guidelines regarding this. Our study indicated that a larger difference between Limbal-D and Lens-D might induce less corneal vertical coma. Jinabhai et al. [30] reported that the less the differences between back optic zone radii and rigid gas permeable (RGP) lens diameter were, the lower the induction of vertical coma component would be. Compared with RGP lenses (nonreverse geometry), OK lenses induced a remodeling of the cornea from prolate to an oblate shape. To clarify the potential mechanism of this phenomenon, we correlated the eccentricity with the ΔLLD and found a positive correlation between them. Based on this finding, we could deduce that the appropriate size of the OK lens may cause less vertical coma. To the best of our knowledge, this is the first time that the association of ΔLLD and vertical coma was found, it will help to improve our current understanding of how OK lens fitting affects HOAs.

Wavefront measurements can result in misinterpretation aberrations and consequently an error between corneal and internal balance because of a technological limitation; these devices exclusively perform pupil-based aberration measurements, rather than vertex-centered measurements like the topographers used to do. The ray-tracing method of iTrace uses a laser beam parallel to the line of sight through the pupil, which measures the exact location where the laser beam reaches the retina by means of the retro-reflected light captured by reference lineal sensors. In this way, a connection is obtained between the direction that the light beams have taken while entering and leaving, allowing a reconstruction of the real wavefront error. It is more physiological to measure these anterior aberrations as the natural trajectory of the light in the eye is analyzed.

In conclusion, we reviewed the profile of each component of WAs during a one-month OK treatment. The ocular WAs induced by OK were mainly attributed to the increase in corneal WAs. The vertical coma (Z3^−1^) of the internal component showed a compensative effect on the corneal vertical coma. Thus, the increase in ocular HOAs after OK is mainly attributed to the increase in horizontal coma (Z3^1^) and SAs (Z4^0^) of the cornea. The degree of myopic correction and subsequent increase in corneal curvature radius may be the essential influential factors of the increase in Z3^1^ and Z4^0^. The appropriate size of the OK lens may be helpful to decrease OK-induced vertical coma. The impact of OK-induced HOAs on visual quality is important and needs further investigation.

## Figures and Tables

**Figure 1:**
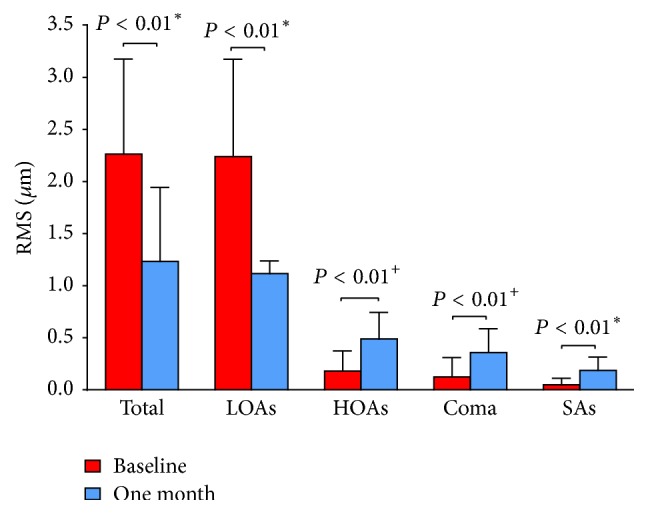
Mean values of root mean square (RMS) in *μ*m of total aberrations (Total), lower-order aberrations (LOAs), higher-order aberrations (HOAs), and coma and Spherical aberrations (SAs) for a 4 mm pupil diameter at baseline and one month after orthokeratology wearing. ^*∗*^Paired *t* test; ^+^Wilcoxon signed-rank test; *n* = 30 eyes.

**Figure 2:**
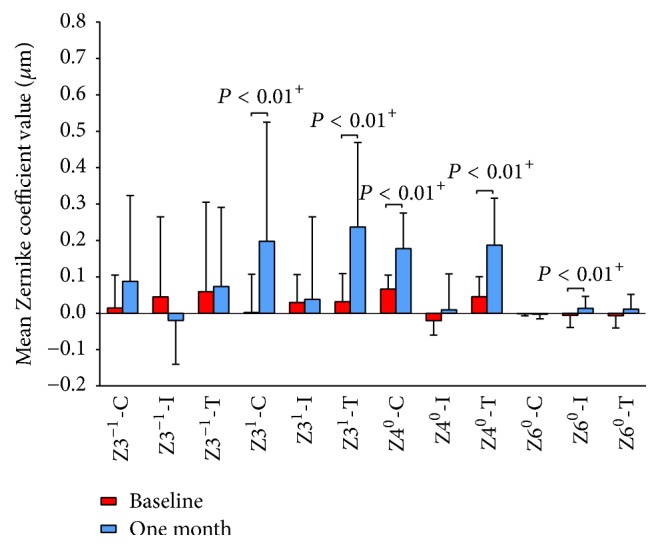
Zernike coefficients (*μ*m) of cornea (-C), internal (-I) and the total (-T) for 4 mm pupil diameter at baseline and one month after orthokeratology wearing; ^+^Wilcoxon signed-rank test; *n* = 30.
